# Understanding Health Information Seeking on the Internet Among Sexual Minority People: Cross-Sectional Analysis From the Health Information National Trends Survey

**DOI:** 10.2196/publichealth.7526

**Published:** 2017-06-19

**Authors:** Jennifer M Jabson, Joanne G Patterson, Charles Kamen

**Affiliations:** ^1^ University of Tennessee Department of Public Health Knoxville, TN United States; ^2^ University of Rochester Cancer Control Unit Department of Surgery Rochester, NY United States

**Keywords:** sexual orientation, Internet-based health information seeking, internet access, sexual minority, homosexuality, bisexuality

## Abstract

**Background:**

Individuals who face barriers to health care are more likely to access the Internet to seek health information. Pervasive stigma and heterosexism in the health care setting are barriers to health care for sexual minority people (SMP, ie, lesbian, gay, and bisexual people); therefore, SMP may be more likely to use the Internet as a source of health information compared to heterosexual people.

**Objective:**

Currently, there is a dearth of published empirical evidence concerning health information seeking on the Internet among SMP; the current project addresses this gap.

**Methods:**

Data from the 2015 Health Information National Trends Survey Food and Drug Administration Cycle were used to describe and summarize health information seeking among SMP (n=105) and heterosexual people (n=3405).

**Results:**

Almost all of the SMP in this sample reported having access to the Internet (92.4%, 97/105). SMP were equally as likely as heterosexual people to seek health information on the Internet (adjusted odds ratio [aOR] 0.94, 95% CI 0.56-1.66) and to report incidental exposure to health information online (aOR 1.02, 95% CI 0.66-1.60). SMP were 58% more likely to watch a health-related video on YouTube than heterosexual people (aOR 1.58, 95% CI 1.00-2.47). Incidental exposure to health information was associated with seeking health information for oneself (aOR 3.87, 95% CI 1.16-14.13) and for someone else (aOR 6.30, 95% CI 2.40-17.82) among SMP.

**Conclusions:**

SMP access the Internet at high rates and seek out health information online. Their incidental exposure could be associated with seeking information for self or others. This suggests that online interventions could be valuable for delivering or promoting health information for SMP.

## Introduction

The general population increasingly uses the Internet as a source of health information. According to the Pew Internet and American Life Project, 88% of the US population has access to the Internet [1] and 61% of adults use the Internet to seek health information [2]. Individuals who engage in online health information seeking for self and others are also more likely to experience incidental exposure to health information [3,4]. Incidental exposure to health information—“information gathered incidentally from sources in the environment” [5]—is worth consideration because it is associated both with increased health knowledge independent of active health information seeking behavior [4], and with positive health behaviors, such as fruit/vegetable intake, exercise, smoking, and cancer screening [5]. According to the Health Belief Model [6], both seeking health information and incidental exposure to health information on the Internet could increase an individual’s perceived susceptibility and perceived threat of disease, which could result in subsequent changes in health behavior and seeking of health care from a provider.

Not all subgroups of the general population use the Internet to seek health information at the same rate nor in the same way [7-10]. Individuals who face educational, economic, and cultural barriers to contact a health care professional are more likely to use the Internet to seek health information and to inform their health care decision making [8,9]. Sexual minority people (SMP; ie, lesbian, gay, and bisexual people) are a group of individuals who face multiple barriers to contact with health professionals. Therefore, it is possible that they may access the Internet at higher rates than heterosexual people (HP) to seek health information.

Pervasive heterosexism and stigma in the form of minority stressors [11,12] have been documented in the health care setting [13-17], and they produce multiple interpersonal and structural barriers for SMPs in accessing care and contacting health care professionals. For example, compared to HP, SMP are 50% less likely to receive needed preventive care including preventive screenings [18,19] and 85% more likely to leave a health care encounter with unmet needs [20]. SMP also report feeling disrespected by their medical providers. Among sexual minority men, 15% report not having enough time with providers, compared to 7% of heterosexual men [21]. In addition, providers also express discomfort and deny the importance of patient’s disclosure of sexual orientation, with 44-63% of health care providers reporting being unaware of SMP in their health care practice [14,22-24]. Health care providers’ medical education does not typically include thorough training in the unique health care needs of SMP, and providers often lack knowledge about health disparities experienced by SMP [25,26].

The Institute of Medicine [27], Healthy People 2020 [28], and the National Institute on Minority Health and Health Disparities [29] have all called for innovative, multilevel, public health solutions to reduce and eliminate health disparities among SMP. Promoting health information access or delivering behavioral health interventions on the Internet targeted to SMP may have multiple health-related benefits. Individuals who seek health information on the Internet are more likely to seek health care professionals for necessary treatments, make informed health care decisions, have positive feelings about information received from a health care professional, and report reductions in risky health behaviors [30,31]. Thus, the Internet has been identified as a promising channel for intervention delivery in the general population, and if SMPs are using the Internet to seek health information, the Internet could also be a useful channel for delivering innovative, disparities-reducing, public health interventions to SMP.

Seeking health information on the Internet may be useful for SMP who fear stigma and discrimination in the health care setting. However, seeking health information on the Internet may also have negative consequences. Health information on the Internet may be inaccurate, may produce anxiety, distress, and fear, and may result in further exposure to stigma for SMP. For example, individuals seeking health information on the Internet via a discussion board concerning human immunodeficiency virus could be exposed to discriminatory and other negative comments about SMP.

Currently there is a dearth of published empirical evidence concerning health information seeking and exposure to health information on the Internet among SMP as compared to HP. To the best of our knowledge, there is currently only one published, empirical article that describes health information seeking on the Internet in relation to sexual orientation [32]. Dahlhamer et al [32] used the National Health Interview Survey to estimate patterns in health information seeking on the Internet. In their study, a larger proportion of SMP than HP sought health information on the Internet.

The current project adds to this area by describing the rates at which SMP use the Internet to seek health information; describing incidental media exposure; estimating how SMP use the Internet for seeking health information compared to HP; and associating incidental and seeking health information on the Internet with health behavior, including seeking a health care provider. We hypothesized that (1) SMP would report higher use of the Internet for health information seeking than HP and that SMP incidental exposure would be similar to HP, and (2) incidental exposure to health information would be associated with seeking health information.

## Methods

### Survey Data

Health Information National Trends Survey (HINTS) Food and Drug Administration (FDA) Cycle 2015 data were used for this project [33]. HINTS is a nationally representative survey of adults aged 18 and older in the civilian, non-institutionalized population, administered by the National Cancer Institute [33]. HINTS was created to monitor changes in health communication and to understand how adults use communication channels to obtain health information. The response rate for HINTS FDA Cycle 4 was 33.04% [33]. Comprehensive information concerning HINTS FDA Cycle 4 methodology is available through HINTS [33].

In the HINTS FDA Cycle 4, 3738 participated and 3510 provided their sexual orientation. A total of 67 people identified as gay or lesbian, 38 as bisexual, and 3405 as heterosexual. Sexual orientation was missing for 228 participants. Due to small sample sizes and to facilitate analyses, sexual orientation was dichotomized where all gay men, lesbians, and bisexual individuals were combined into one group (SMP; n=105) and all heterosexual individuals were a second group (HP; n=3405). We believe this is a valid solution for small sample sizes for underrepresented populations, although we also acknowledge the limitations this may introduce based on documented differences between sexual minority subgroups [27].

### Measures

#### Dependent Variables

Sources and frequency of Internet access were measured with two questions: “Do you ever go online to access the Internet” (yes/no) and “How often do you access the Internet through each of the following: computer at home, computer at work, computer at school in a public place, computer at school in a private place, on a mobile device, on gaming device, Other” (daily, sometimes, never, n/a). Reasons for Internet use were measured with one question: “Sometimes people use the Internet for health-related reasons. Have you used the Internet for any of the following reasons in the past 12 months?” Sample reasons in the survey include “looked for health information for yourself, looked for health information for someone else, looked for information about quitting smoking.” Reponses were dichotomous (yes/no).

#### Independent Variable

Sexual orientation was measured with a single item, “Do you think of yourself as heterosexual or straight, homosexual or gay/lesbian, bisexual, something else?” Sexual orientation was dichotomized (sexual minority=1, heterosexual=0). Respondents’ qualitative descriptions of “something else” did not indicate a sexual minority identity. Many of the responses included statements such as “normal,” “god’s child,” “human.” Therefore, respondents who selected “something else” (n=57) were excluded from the analysis.

Frequency and reading of incidental health information exposure online were measured with two questions: “Some people notice information about health on the Internet, even when they are not trying to find out about a health concern they have or someone in their family has. Have you read such health information on the Internet in the past 12 months?” (yes/no) and “About how often have you read this sort of information in the past 12 months?” (once a month or more, less than once a month).

#### Demographic Characteristics and Covariates

Demographic characteristics included age at time of survey, race/ethnicity (Hispanic, non-Hispanic Black/African-American, non-Hispanic white, other; ref=non-Hispanic white), highest level of education achieved (less than high school, high school/General Equivalency Diploma, some college/Associate degree, college graduate/above; ref=less than high school), gender (male/female), insurance coverage (insured/uninsured), and income.

Time spent using the Internet for personal reasons was measured with two variables. First, “On a typical weekday, about how many hours do you use the Internet for personal reasons?” Respondents self-reported the number of hours on a typical weekday. Second, “During a typical weekend, about how many hours do you use the Internet for personal reasons?” Respondents self-reported the number of hours on a typical weekend.

### Analyses

Descriptive statistics were calculated to describe and summarize demographic characteristics and covariates, sources and frequency of Internet access, and reasons for health information seeking among SMP. Chi-square tests were calculated for categorical variables for differences by sexual orientation. The *t* test was calculated to test for difference in continuous variables including age and average number of hours individuals accessed the Internet on weekdays and the weekend, by sexual orientation. Demographic characteristics that varied significantly by sexual orientation were applied as adjustment variables for multivariable tests. Multiple logistic regressions were calculated to test for association between sexual orientation and dependent variables. All of the multivariable logistic regression analyses were adjusted for age (continuous), education (categorical), and race/ethnicity (categorical); dummy variables were created for each categorical variable. Models were adjusted to account for possible confounded association between sexual orientation and health information seeking on the Internet. All adjustment variables were entered into each of the multivariable logistic regression models simultaneously. Multivariable logistic regression models concerning incidental exposure were also adjusted for average number of hours spent using the Internet on typical weekdays and weekends. Analyses were conducted with SPSS 14.0. This secondary analysis did not include human subjects and did not require a human subjects review.

## Results

The sample’s demographic characteristics are stratified by sexual orientation and summarized in Table 1. SMP were more likely to be Hispanic (χ^2^_3_=13.91, *P*=.004), younger (*t*_1_=4.39, *P*<.001), and to have achieved higher levels of education (χ^2^_3_=8.10, *P*=.040) than HP.

**Table 1:** Demographic characteristics by sexual orientation.

Characteristics		n (%) or mean (SD)			X^2^/ *t*	df	*P*
		Full	Sexual minority	Heterosexual			
Total		3738 (100.0)	105 (3.0)	3405 (97.0)			
**Sex**					2.50	1	.13
	Male	1497 (42.6)	52 (50.5)	1394 (42.7)			
	Female	2018 (57.4)	51 (49.5)	49.5 (57.3)			
**Race/Ethnicity**					13.91	3	<.01
	Hispanic	241 (7.2)	16 (15.2)	201 (6.4)			
	White, Non-Hispanic	2633 (78.2)	72 (68.6)	2479 (79.1)			
	Black or African-American, Non-Hispanic	232 (6.9)	6 (5.7)	212 (6.8)			
	Other, Non-Hispanic	260 (7.7)	7 (6.7)	243 (7.8)			
Age		61.1 (17.5)	47.9 (15.6)	56.8 (16.6)	5.41	3508	<.001
**Education**					8.10	3	.04
	<High school	237 (6.5)	5 (4.8)	194 (5.8)			
	High school/GED	727 (19.8)	11 (10.5)	664 (19.5)			
	Some college/AA degree	1132 (30.8)	30 (28.6)	1045 (31.0)			
	College graduate or above	1578 (43.0)	58 (55.2)	1466 (43.1)			
**Household income**					1.97	4	.74
	<$20,000	664 (20.1)	16 (15.7)	591 (19.1)			
	$20,000-$34,999	506 (15.3)	13 (12.7)	473 (13.9)			
	$35,000-$49,999	415 (12.6)	14 (13.7)	392 (12.7)			
	$50,000-$74,999	605 (18.3)	23 (22.5)	573 (18.6)			
	>$75,000	1112 (33.7)	36 (35.3)	1061 (34.3)			
**Insurance coverage**					0.01	1	1.00
	Uninsured	207 (5.7)	6 (5.7)	186 (5.5)			
	Insured	3444 (94.3)	99 (94.3)	3197 (94.5)			
**Smoker**					1.83	2	.41
	Never	2041 (55.6)	51 (50.5)	1849 (55.3)			
	Current	495 (13.5)	18 (17.8)	448 (13.4)			
	Former	1132 (30.9)	32 (31.7)	1049 (31.4)			

Unadjusted descriptive statistics for sources and frequency of Internet access by sexual orientation are presented in Table 2. SMP (92.4%, 97/105) were more likely to report Internet access than HP (79.4%, 2702/3738; χ^2^_1_=10.64, *P*=.001). More SMP (26.1%, 24/105) reported accessing the Internet on a computer at school in a public location than HP (15.5%, 396/3738; χ^2^_1_=7.37, *P*=.009).

Table 2 also presents the unadjusted frequencies and descriptive reasons for seeking health information on the Internet, stratified by sexual orientation. SMP most frequently used the Internet to seek health information for themselves (88.1%, 77/105), followed by seeking health information for someone else (60.0%, 57/105), and keeping track of personal health information (54.3%, 51/105). Watching health-related videos on YouTube was the only difference between SMP and HP in reasons for seeking health information on the Internet. More SMP watched health-related videos on YouTube (37.2%, 35/105) than HP (22.5%, 593/3405; χ^2^_1_=11.21, *P*<.001).

**Table 2:** Sources, frequency, reasons, and unintentional exposure to health information via Internet access and use by sexual orientation.

Sources and frequency of Internet access			n (%) or mean (SD)		X^2^/ *t*	df	*P*
			Sexual minority	Heterosexual			
Accesses Internet			97 (92.4)	2702 (79.4)	10.64	1	<.001
**Average number of hours accessed Internet**							
	Weekday		4.18 (4.32)	2.79 (3.52)	-3.21	105	<.01
	Weekend		4.92 (3.90)	3.69 (4.37)	-2.81	3380	<.01
**Where accesses Internet**							
	Computer at home		90 (95.7)	2417 (92.5)	1.42	1	.32
	Mobile device		75 (81.5)	1984 (76.5)	1.26	1	.32
	Computer at work		54 (58.7)	1269 (49.8)	2.82	1	.11
	Computer at school, public space		24 (26.1)	396 (15.5)	7.37	1	.01
	Gaming device		21 (22.8)	397 (15.5)	3.55	1	.08
	Computer at school, private space		12 (13.3)	201 (8.0)	3.26	1	.08
	Other		7 (9.2)	105 (4.9)	2.88	1	.10
Noticed and read health information on Internet			62 (59.0)	1562 (45.9)	1.63	1	.21
**How often read health information online**						1	
	Once a month		40 (65.6)	1000 (64.9)	0.01		1.00
	Less than once a month		21 (34.4)	541 (35.1)	0.01		1.00
Used Internet last time sought health information			74 (89.2)	1669 (68.6)	15.97	1	<.001
Contacted doctor or health care provider last time sought health information			3 (3.6)	426 (17.5)	10.95	1	<.001
**Reasons for Internet use**							
	Seeking health information for self		77 (88.1)	2076 (78.3)	0.41	1	.53
	Seeking health information for someone else		57 (60.0)	1728 (65.3)	1.12	1	.32
	Kept track of personal health information		51 (54.3)	1257 (47.5)	1.68	1	.21
	Exchanged support about health concerns with family/friends		49 (52.1)	1237 (46.7)	1.06	1	.34
	Used website to help with diet, weight, or physical activity		47 (49.5)	1163 (44.0)	1.13	1	.30
	Seeking health care provider		42 (44.7)	943 (35.9)	3.02	1	.10
	Watched a health-related video on YouTube		35 (37.2)	593 (22.5)	11.21	1	.001
	Shared health information on social media sites		17 (18.1)	391 (14.8)	0.79	1	.38
	Downloaded health information		14 (14.7)	397 (15.0)	0.00	1	1.00
	Seeking information about quitting smoking		9 (9.6)	160 (6.1)	1.93	1	.18
	Participation in online forum/support group		4 (4.2)	135 (5.1)	0.15	1	.82
**Frequency of unintentional health information seeking**							
	Unintentionally noticed health information		62 (59.0)	1562 (58.0)	1.63	1	.21
	**How often read this type of information**					1	
		Once a month or more	40 (65.6)	1000 (64.9)	0.01		1.00
		Less than once a month	21 (34.4)	541 (35.1)	0.01		1.00

Frequency of incidental exposure to health information (SMP 59% vs HP 58%) and frequency of reading such information did not vary by sexual orientation (Table 2). On a typical weekday, SMP and HP used the Internet for personal reasons on average 4.18 (SD 4.32) and 2.79 (SD 3.52) hours respectively (*t*_105_*=*-3.21, *P*<.01). Internet use for personal reasons on a typical weekend also varied by sexual orientation. On average, SMP reported 4.92 (SD 3.90) and HP 3.69 (SD 4.37) hours (*t*_3380_=-2.81, *P*<.01).

Table 3 presents the adjusted associations between access to Internet and reasons for seeking health information on the Internet. In analyses adjusted for age, education, and race/ethnicity, SMP were 38% less likely than HP to report seeking health information on the Internet for someone else (adjusted odds ratio [OR] 0.62, 95% CI 0.40-0.97; *P*=.03). After adjusting for age, race/ethnicity, and education, SMP were 58% more likely to report watching health-related videos on YouTube compared to HP (aOR 1.58, 95% CI 1.00-2.47; *P*=.04).

**Table 3:** Associations^a^ between sexual orientation and health information seeking behaviors.

	Sexual minority (n=105) (heterosexual ref)	
	aOR (95% CI)	*P*
Accessed Internet	1.84 (0.85-4.56)	.15
Noticed health information (incidental exposure)^b^	1.02 (0.66-1.60)	.92
Read health information once a month or more (incidental exposure)^b^	0.83 (0.48-1.47)	.51
Seeking health information for self	0.94 (0.56-1.66)	.83
Seeking health information for someone else	0.62 (0.40-0.97)	.03
Kept track of personal health information	1.28 (0.84-1.96)	.25
Exchanged support about health concerns with family/friends	1.17 (0.77-1.78)	.47
Used website to help with diet, weight, or physical activity	0.95 (0.62-1.47)	.82
Seeking health care provider	1.14 (0.74-1.76)	.54
Watched a health-related video on YouTube	1.58 (1.003-2.47)	.04
Shared health information on social media sites	1.01 (0.56-1.72)	.97
Downloaded health information	0.76 (0.40-1.33)	.36
Seeking information about quitting smoking	1.37 (0.62-2.60)	.39
Participation in online forum/ support group	0.70 (0.21-1.72)	.50

^a^All models adjusted for age, education, and race/ethnicity.

^b^Model adjusted for age, education, race/ethnicity, and average number of weekday and weekend hours accessed Internet.

Associations between incidental exposure to health information on the Internet and health information seeking behaviors were calculated with adjustment for age, race/ethnicity, and education, and stratified by sexual orientation (data available on request). Among HP, incidental exposure to health information on the Internet was associated with all health information seeking behaviors. Among SMP, small sample sizes made it impossible to calculate adjusted analyses for several models including seeking a health care provider, watching a health-related video, downloading health information, and seeking information about quitting smoking. For the models that could be calculated, incidental exposure to information online was associated with three times greater odds of seeking health information for self (aOR 3.87, 95% CI 1.16-14.13; *P*<.05) and six times greater odds of seeking health information for someone else (aOR 6.30, 95% CI 6.30-17.82; *P* ≤.001), relative to those who did not report incidental exposure to health information on the Internet.

## Discussion

### Principal Findings

The purpose of this project was to describe SMP’s access to the Internet and to investigate SMP health information seeking and incidental exposure to health information on the Internet. Our results indicate that 94.4% of SMP respondents are accessing the Internet, and adjusted analyses indicate that this was not significantly different from heterosexual respondents.

We found that 88.1% of SMP and 78.3% of HP are seeking health information on the Internet for themselves. These findings are similar to those published by Dahlhamer et al [32]. From data provided by the National Health Interview Survey, Dahlhamer et al reported that 62.3% of sexual minority men and 65.8% of sexual minority women sought health information on the Internet compared to 42.3% of heterosexual men and 56.2% of heterosexual women. SMP have access to the Internet and are using the Internet to seek health information. This evidence is especially valuable for public health practitioners and researchers interested in testing and disseminating Internet-based interventions for improving health and reducing disparities among SMP [34].

SMP sought health information on the Internet for reasons that were largely the same as HP. One notable difference was in access to health-related videos on YouTube, where SMP were more likely to report viewing health-related videos than HP. Social media and YouTube have been used by some public health interventionists as a mechanism to reach gay and bisexual men for sexual health promotion [35,36]. In their review of social media for sexual health promotion, Gabarron and Wynn [36] found eight projects that determined that YouTube would be an effective means of delivering intervention content to SMP. Many of these projects reported thousands of online views, including “Queer as F**ck,” which delivered sexual health promotion to gay and bisexual men via short “webisodes” [35]. Such webisodes were highly popular and commanded over 30,000 YouTube views. These and other forms of sexual health promotion underscore the potential utility of YouTube for promoting health messages among SMP. It is also possible that SMP may be using health-related videos in response to experienced or anticipated barriers in accessing health care. Given the breadth of evidence documenting SMPs’ experiences of heterosexism and homophobia in the health care setting [13-17], it may be that in the absence of consistently accessible, culturally competent care, SMP are more likely to seek health information from online sources, such as YouTube, that reflect their specific sexual orientation group or target relevant health concerns [37].

There are also methodological issues that could have influenced the results involving incidental exposure. The sample of SMP was small, and it may have been too small to detect an association. This is something that can be addressed only by increasing sample sizes of SMP in health surveillance. With respect to the significant associations identified between incidental exposure and health information seeking activities, ours was a cross-sectional, secondary study. Therefore, it cannot be known if incidental exposure came before, or after, seeking health information for self and seeking health information for someone else. It is plausible that in the process of seeking health information for themselves or someone else, SMP were incidentally exposed to health information online. This is a research question to be addressed by future research efforts that can test temporality.

It is useful to know that SMP are being exposed to incidental health information for the purposes of future health information programs that aim to target SMP. It could be useful for future Internet-based health information programs to know the types of health information that SMP are the most likely to read, and future studies should assess the health information to which SMP are exposed with greater nuance.

### Strengths and Limitations

HINTS is a population-based data source focused on Internet access and health information seeking on the Internet that also includes sexual orientation questions. As a result, this project represents some of the first population-based evidence on Internet access among and health information seeking on the Internet among SMP. A very small number of individuals did not answer the sexual orientation question, suggesting that the population is increasingly comfortable reporting their sexual orientation in health surveillance. Sexual minority samples sizes are notoriously small, with some population-based findings based on 50 or fewer SMP. Although still relatively small, HINTS provided a sample of more than 100 SMP, equally male and female.

This project involved a small sample of SMP. Small sample sizes result in underpowered statistical analyses and thereby make it difficult to detect statistically meaningful differences if differences between SMP and HP are present. Small sample size is a persistent problem when using health surveillance data sources to investigate SMP health. In order to capture the best possible approximation of a representative sample, the Institute of Medicine [27] and other national organizations strongly encourage the use of health surveillance data sources, such as HINTS, to investigate sexual minority health. However, this approach often produces very small samples where only 3-5% of the total sample comprising SMP, and sometimes much less. One possible solution for this in the future could be rigorous oversampling of SMP in health surveillance. The HINTS response rate is low (33.04% [33]) and may reflect the healthy volunteer effect where only the healthiest individuals participate [38]. Finally, our statistical methods that involved multiple tests of associations may have also put the findings at risk of a type 1 error in which we reported associations that were produced by chance. In addition, the confidence intervals and *P* values for “seeking health information for self” and “accessed the Internet” are very near and barely including 1.0 with *P* values of .06 and .05. It is possible that these confidence intervals and *P* values are an artifact of our statistical software package and small sample sizes. We believe that replicating these analyses with larger samples would aid in clarifying these limitations and associations.

### Conclusion

It is often difficult to locate sexual minority people for health-related, disparities-reducing interventions, but our findings suggest that the Internet is a promising tool for delivering health interventions to this group. SMP use the Internet and are using it to access health information at high rates. This is valuable given the popularity and promise of Internet-based interventions for SMP. We now have evidence that the Internet is a promising delivery method for health-related information for SMP.

## Abbreviations

aOR: adjusted odds ratio
HINTS-FDA: Health Information National Trends Survey Food and Drug Administration
HP: heterosexual people
SMP: sexual minority people
